# Natural Triterpenic Diols Promote Apoptosis in Astrocytoma Cells through ROS-Mediated Mitochondrial Depolarization and JNK Activation

**DOI:** 10.1371/journal.pone.0005975

**Published:** 2009-06-22

**Authors:** Rubén Martín, Elvira Ibeas, Juliana Carvalho-Tavares, Marita Hernández, Valentina Ruiz-Gutierrez, María Luisa Nieto

**Affiliations:** 1 Instituto de Biología y Genética Molecular, CSIC-Universidad de Valladolid, Valladolid, Spain; 2 Instituto de la Grasa de Sevilla (CSIC), Sevilla, Spain; Bauer Research Foundation, United States of America

## Abstract

**Background:**

Triterpene alcohols and acids are multifunctional compounds widely distributed throughout the plant kingdom that exhibit a variety of beneficial health properties, being synthetic analogs of oleanolic acid under clinical evaluation as anti-tumoral therapeutic agents. However, the antineoplastic activity of two natural occuring triterpenoid alcohols extracted from olive oil, erythrodiol (an intermediate from oleanolic acid), and its isomer, uvaol, has barely been reported, particularly on brain cancer cells. Astrocytomas are among the most common and aggressive type of primary malignant tumors in the neurological system lacking effective treatments, and in this study, we addressed the effect of these two triterpenic diols on the human 1321N1 astrocytoma cell line.

**Principal Findings:**

Erythrodiol and uvaol effectively affected cell proliferation, as well as cell cycle phases and induced 1321N1 cell death. Both triterpenes successfully modulated the apoptotic response, promoting nuclear condensation and fragmentation. They caused retraction and rounding of cultured cells, which lost adherence from their supports, while F-actin and vimentin filaments disappeared as an organized cytoplasmic network. At molecular level, changes in the expression of surface proteins associated with adhesion or death processes were also observed. Moreover, triterpene exposure resulted in the production of reactive oxygen species (ROS) with loss of mitochondrial transmembrane potential, and correlated with the activation of c-Jun N-terminal kinases (JNK). The presence of catalase reversed the triterpenic diols-induced mitochondrial depolarization, JNK activation, and apoptotic death, indicating the critical role of ROS in the action of these compounds.

**Conclusions:**

Overall, we provide a significant insight into the anticarcinogenic action of erythrodiol and uvaol that may have a potential in prevention and treatment of brain tumors and other cancers.

## Introduction

Improvements in current cancer treatment regimens have resulted in an increase in patient survival. Nevertheless, many tumors, particularly astrogliomas, the most common primary brain tumors, relapse and are resistant to subsequent treatments. Several obstacles prevent the complete eradication of these high-grade malignant neoplasms by conventional therapies: i) their ability to infiltrate the surrounding normal brain, rendering total surgical excision highly improbable [1], ii) their low response or resistance to chemotherapy, and specially iii) their physical isolation from systemic circulation due to the impermeability of the blood–brain barrier (BBB), which limits transport of most hidrophilic and large lipophilic molecules, thus preventing more than 95% of drugs from penetrating the brain [2]. Therefore, search new drugs or how to get these drugs across the natural guardian of the brain, the BBB, is the cutting edge research to find solutions to these highly invasive tumors.

Accumulating data indicate that the cytotoxic effect of many chemotherapeutic drugs occurs through programmed cell death (apoptosis) [3]. Hence, the ability of tumor cells to respond and activate the apoptotic program may, in part, determine the success of the therapeutic strategy [4]. It is well documented that apoptosis can be induced by a variety of drugs with diverse chemical structure and different mechanism of action; and two major routes including the death-receptor pathway and the mitochondrial-pathway have been identified [5]. Apoptosis is a highly regulated process that involves many proteins and genes [6], [7]. It is characterized by cell shrinkage, plasma membrane bebbling, and chromatin condensation. The death program is executed by caspases, which amplify the apoptotic signal and proteolytically process numerous cellular molecules with different functions [8]. In response to death stimuli, ROS accumulation and alterations in the mitochondrial membrane potential (ΔΨ_m_) are considered to be early events [9]. In addition, mitogen-activated protein kinases (MAPK) have also been considered as an upstream signal for the initiation of apoptosis. Many studies have shown that the stress-activated protein kinases pathway are rapidly activated in response to oxidative insults and are frequently associated with cell death. Thus, their activation is usually correlated with apoptosis induced by agents that act, at least in part, via ROS generation [10]–[13].

A critical genetic defect in many tumors, including gliomas, is found in the p53 gene, which makes p53 non-functional. Normally, p53 sensitizes the tumor cell to chemotherapy, which will induce programmed cell death. Thus, the search for effective agents to treat a broad spectrum of tumors that differ in the expression levels or in the mutational status of p53, are objectives of drug development programs. At present, it has been reported that members of the natural occurring triterpene family such as boswellic, maslinic, ursolic or betulinic acid among others, are potent apoptotic agents to cancer cells regardless of their p53 status (null, wild-type or mutant) [14], [15].

Triterpenes are compounds widely available in fruits and vegetables in human diet, as in the components of natural herbal preparations used for the treatment of human diseases. Chemically pentacyclic triterpenes are all based on a 30-carbon skeleton comprising five six-membered rings (ursanes and oleananes) or four six-membered rings and one five-membered ring (lupanes), and as lipophilic molecules may also penetrate the blood-brain barrier, as it has already been demonstrated for some of them. [16]–[18]. The plant *Olea europaea*, the origin of the cultivated olive, is widespread in Mediterranean countries, and extracts of its leaves, flowers and fruits possess therapeutic properties and have been used traditionally for medicinal purposes [19]–[22]. Among the major components present in the unsaponifiable fraction of olive-pomace oil are the triterpenic acids, oleanolic and maslinic acids and the triterpenic diols, uvaol and erythrodiol [23]. Many pharmacological properties of these compounds have been reported. Thus, antiparasitic [24], hepatoprotective [25], anti-VIH [26], anti-inflammatory and antioxidant activities [27], [28] have been attributed to them. In addition, recent “in vivo” studies have demonstrated the benefits of oleanolic acid in preventing hypertension [29]. Indeed, oleanolic acid, as well as uvaol and the maslinic acid derivative, methyl maslinate, have also been shown to possess vasodepressor, cardiotonic, and antidysrhythmic properties [30]. Furthermore, they are able to induce vasorelaxation in the aorta of hypertensive rats [31]. And recently, it has been suggested that erythrodiol derivatives can prevent the harmful effects of ultraviolet rays that lead to skin aging or skin cancer [32].

In the course of the search for potential antitumor promoters from natural sources, the anticancer effect of these triterpenes started to draw attention. Several studies have suggested that both acidic and alcoholic triterpenes present anti-tumor activities [33], [34]. Although they markedly differ in their cytotoxic activity and the precise mechanism of action is still unclear, especially for the triterpenic diols. Thus, oleanolic and maslinic acids are powerful pro-apoptotic agents in human colon cancer cells [35], [36], while uvaol show weak activity against an array of human cancer cell lines from different tissues [34]. However, although in a recent study we found that oleanolic and maslinic acids are potent inducers of apoptosis in astroglioma cells, to date, there is still little data available regarding their effects, as well as of the rest of the natural triterpenoids, in brain cancer cells [37]. Therefore, we propose to examine on astroglial tumor cells, the anti-proliferative and pro-apoptotic activities of the erythrodiol, an intermediate of oleanolic and maslinic acid, and its isomer, the ursane diol uvaol, as well as to elucidate the role of ROS in the anti-neoplasic activities of these promising bioactive compounds.

## Results

### Effect of erythrodiol and uvaol on 1321N1 cell attachment

**Figure 1:**
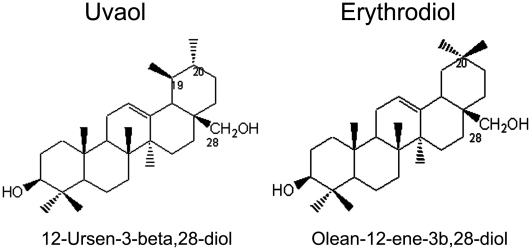
Molecular structure of uvaol and erythrodiol.

First of all, commercial erythrodiol and uvaol (Figure 1) were assessed for purity and identity by GC/MS (Figure S1), a method that is based on the derivatisation of these molecules by silylation with TMSIM as recommended procedure. Figure S1A and C show the chromatographic peaks of the uvaol and erythrodiol samples with retention times at 30.7 and 29.7 minutes, respectively. The peak purity was of 99%, which can be considered a pure compound. Chromatograms B and D, show the corresponding mass spectra of the silylated molecules, which were in close agreement with those fragmentation patterns reported in the literature for these compounds (Wiley Registry of Mass Spectral data base).

**Figure 2:**
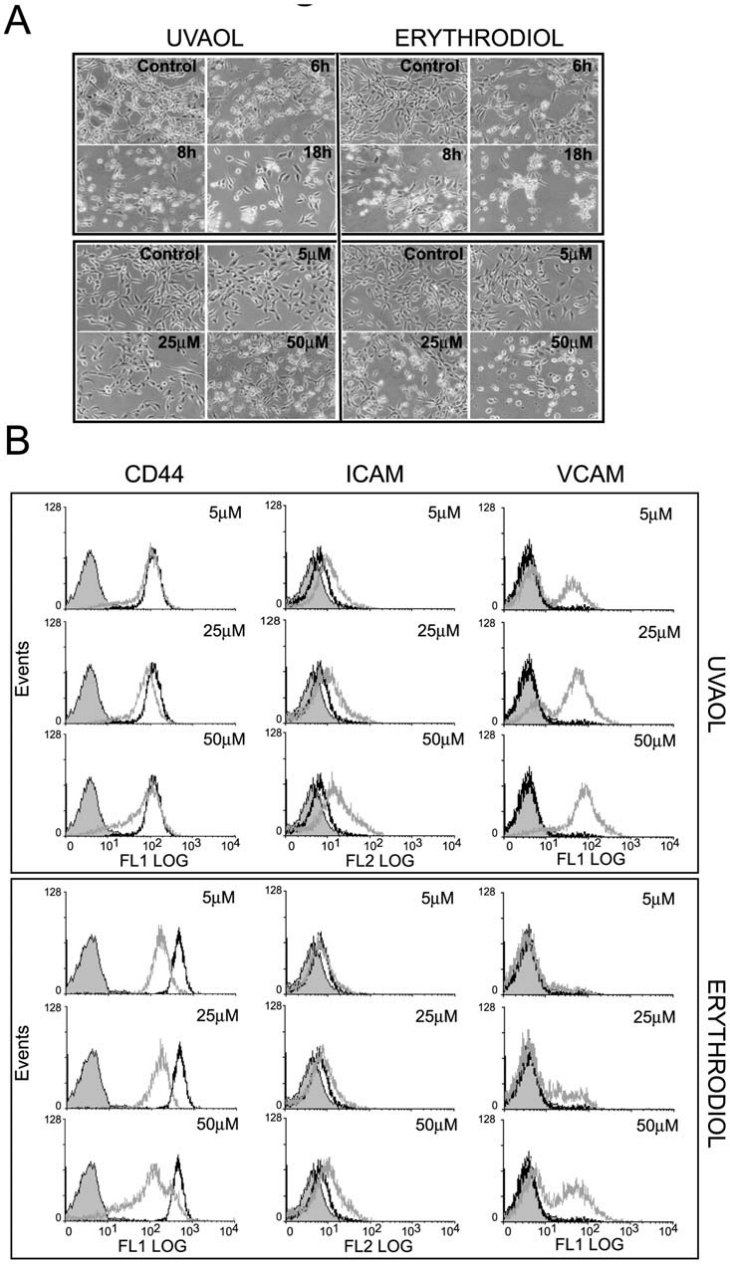
Erythrodiol and uvaol induce detachment on 1321N1 astrocytoma cells. A, Morphological appearance of the cells exposed to different doses of erythrodiol or uvaol for 6 h (*upper panels*) or to 25 µM erythrodiol or 50 µM uvaol for different times (*lower panels*). Cells were visualized under a phase contrast microscope Nikon Eclipse TS100 (×20). B, Cells were treated with different doses of erythrodiol or uvaol for 18 h. The expression of CD44, ICAM and VCAM was determined by flow cytometry. Histograms represent one experiment out of three. *Solid gray* curves represent unspecific binding; *empty black* curves, cells cultured in the absence of treatment (control); and *empty gray* curves, triterpene-treated cells.

After that, we moved on to characterize their biological activities. In a primary screening test, we found that the alcoholic triterpenes, erythrodiol and uvaol, were able to modify astrocytoma cell morphology in a dose- and time-dependent manner (Fig. 2). Under the phase-contrast microscope, 1321N1 astrocytes cultured in normal conditions showed an adherent monolayer and a large flat and polygonal shape; however, following 6 h of exposure to different doses of erythrodiol or uvaol, cell morphology changed dramatically. There was a morphological response characterized by cytoplasmic retraction and rounding-up of the cell body, compared to untreated cells (Fig. 2A *upper panels*). Although both triterpenes displayed similar changes, cells were more sensitive to erythrodiol. Thus, the morphological transformation induced by erythrodiol was detectable at 25 µM and maximum at 50 µM, while uvaol response at 25 µM was barely visible. Therefore, the concentrations chosen for the time-course experiments were 25 µM for erythrodiol and 50 µM for uvaol. As shown in Figure 2A (*lower panels*), the rate of morphological transformation was similar for both triterpenes. After 8 h of treatment cells began to detach from the culture flask, and by 18 h were seen in aggregates as clusters of rounded cells.

Next, we studied the modulation of surface molecules such as ICAM-1, VCAM-1 or CD44, since alterations in their expression levels could cause changes in cell-substrate adhesion and/or in homotypic cell-cell contact (Fig. 2B). By flow cytometry analysis, resting 1321N1 cells showed immunofluorescence above unespecific levels with antibodies to ICAM-1 and CD44, but not with antibody to VCAM-1. Incubation for 18 h with different doses of erythrodiol or uvaol, significantly diminished the expression levels of CD44, while VCAM-1 and ICAM-1 immunoreactivity was markedly increased.

### Erythrodiol and uvaol induce morphological changes in 1321N1 cells

**Figure 3:**
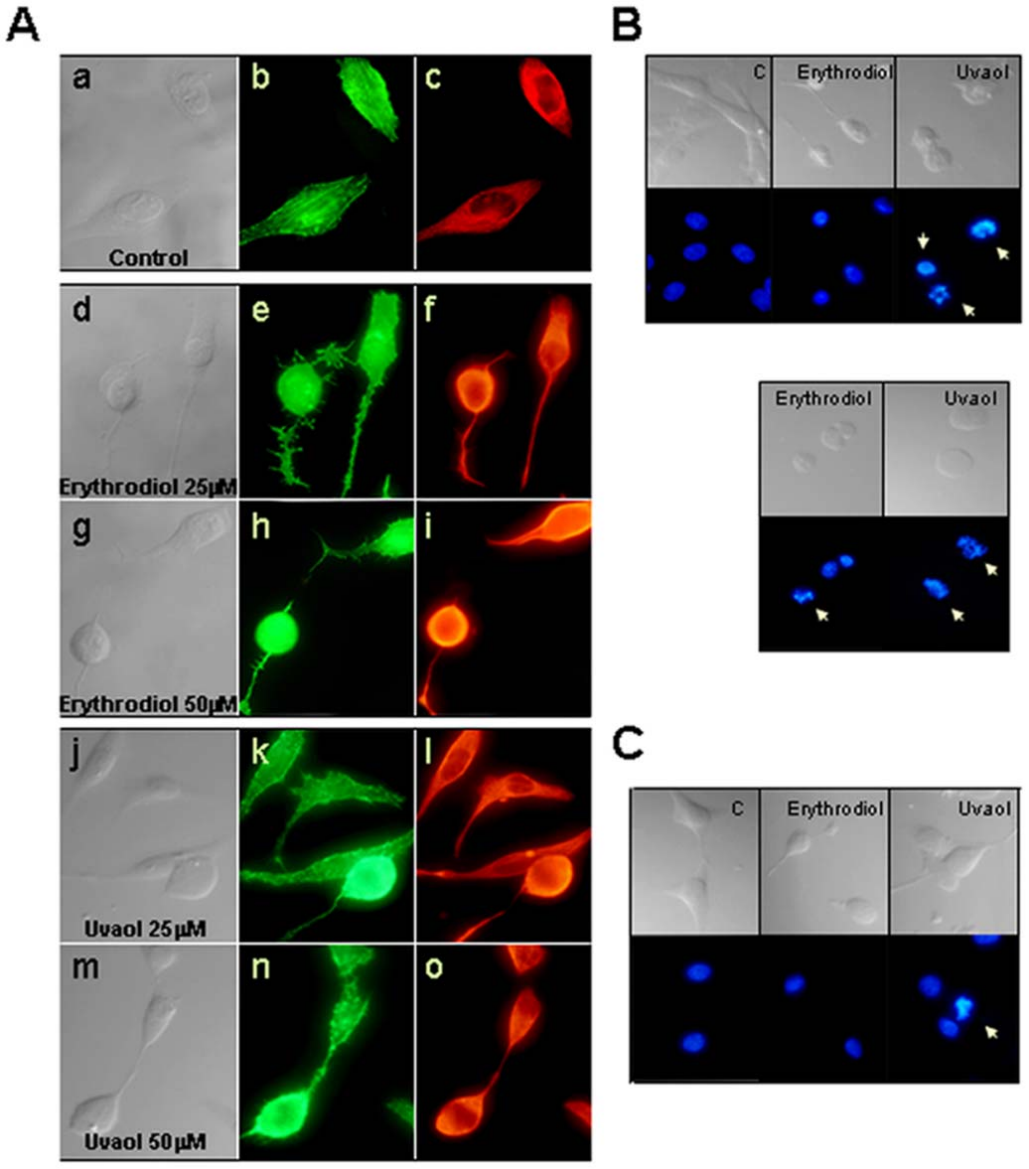
Erythrodiol and uvaol induce morphological changes in the cytoskeleton. A, Cells were treated with different doses of erythrodiol or uvaol for 6 h. Then, cells were stained with FITC-phalloidin (green, b, e, h, k and n) or anti-vimentin Ab (red, c, f, i, l and o) and visualized under a fluorescent microscope (×60). Cells were seeded on standard conditions (B) or in poly-l-lysine treated coverslips (C). After 18 h of exposure to 25 µM of triterpenes, floating cells (B *lower panels*) and attached cells (B *upper panels* and C) were fixed and stained with DAPI. Cells were visualized using a Nikon Eclipse 80i fluorescent microscope (×60). The cellular morphology was observed using Nomarski optics. The floating population from the adherent condition was too low to be evaluated.

Cytoskeletal elements are known to be involved in determining cell shape and cellular contacts. In order to prove that the morphological changes observed in the triterpene-treated astrocytes were associated with alterations in the cytoskeleton, we examined the organization of cytoskeletal proteins, F-actin and vimentin (Fig. 3A). In resting cells, F-actin appeared in a well organized crossing pattern of stress fibbers that traverses the whole cell. When cells were exposed to 25 and 50 µM of erythrodiol or uvaol, this organized system of actin cables looked completely disrupted, being more relevant at the highest doses. This rapid depolymerization and breakdown of actin determined that the stress fibers were no longer evident, and the F-actin staining was found diffuse throughout the cytoplasm and also in the long and thin cellular projections. The staining and distribution pattern of vimentin in triterpenes-treated cells also revealed an altered assembly, following a similar model to that found for F-actin. Control cells displayed vimentin staining as a complex mesh of fibbers, most evident in a perinuclear distribution within the cell body. After triterpene treatment, this network of fibers packed together into a continuous dense bundle along the cellular processes.

Then, to assess whether the cytoskeletal rearrangements were associated to nuclear alterations, we tested for an increase in nuclear/chromatin fragmentation. Cells were seeded in standard tissue culture plates and after 18 h of exposure to triterpenes, although some of the 1321N1 astrocytes kept anchorage onto the coverslips (Fig. 3B *upper panels*), most of them were in suspension (Fig. 3B *lower panels*), so we also collected them for fluorescence microscopy analysis. DAPI staining revealed that in the presence of uvaol, both attached and floating cells showed clear apoptotic features, such as nuclear fragmentation, while after erythrodiol treatment nuclear changes were only found in the detached cells.

Next, to achieve better adhesion settings, cells were seeded onto poly-l-lysine-treated coverslips (Fig. 3C). In these conditions no nuclear fragmentation was detected in erythrodiol treated-cells. Conversely, the presence of uvaol in this adherent situation induced a nuclear morphology characteristic of the apoptotic process.

### Effect of triterpenic alcohols on 1321N1 cell growth and survival

The effect of erythrodiol and uvaol on survival of 1321N1 astrocytoma cells was determined by [^3^H]-thymidine incorporation, PI staining, and annexin-V binding [37], [38].

**Figure 4:**
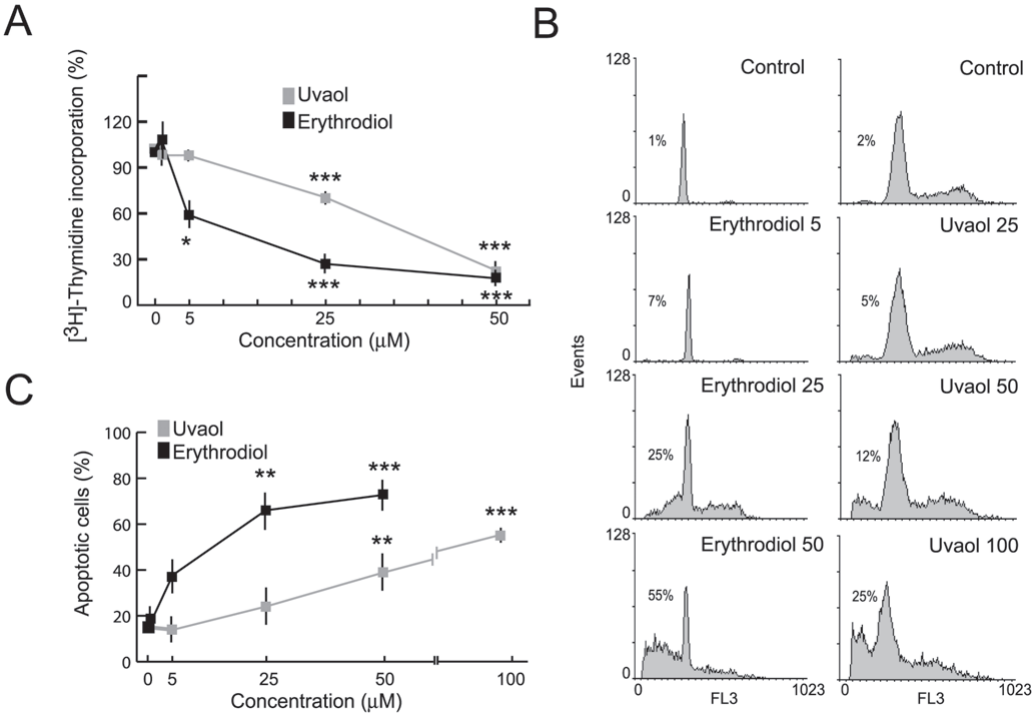
Effects of triterpenic diols on cell growth and apoptosis in 1321N1 cells. A, The cells were exposed to different doses of erythrodiol or uvaol for 18 h in the presence of FCS and proliferation was determined by a [^3^H]-thymidine uptake assay. Data are expressed as the percentage of radioactivity incorporated in FCS-stimulated cells in the absence of triterpenes (327.510±3.889 dpm). B, C, Cells treated as above, but without FCS, were fixed in 70% ethanol and stained with PI (B) or stained with annexin-V-PE (C) and analyzed by flow cytometry. The numerical values are presented as the mean±S.D. of three independent experiments. Percentages in B indicate the number of cells in the sub–G0-G1 phase of the cell cycle. *p<0.05, **p<0.01, ***p<0.001 vs control untreated cells.

The thymidine uptake assay confirmed that DNA synthesis was significantly inhibited in cells treated with 1–50 µM triterpenes (Fig. 4A). The resulting growth curves showed a dose-dependent inhibitory effect. erythrodiol-treatment inhibited 1321N1 proliferation at lower IC50 than uvaol.

Next, the cell cycle distribution was examined under the same conditions (Fig. 4B). A hallmark of apoptosis in the cells is the generation of DNA fragments, which leads to a characteristic hypodiploid pattern, readily distinguishable by flow cytometry analysis after PI staining. Thus, cell cycle analysis revealed a progressive accumulation of cells in the subdipliod phase (sub-G_1_) of the cycle upon exposure to triterpene. Again, the effect with uvaol was smaller than with erythrodiol at the same concentration. Apoptotic rate in erythrodiol- and uvaol-treated cells increased in a dose-dependent manner, from 1.1% to 55.3%, and from 2% to 35%, respectively. Moreover, another apoptotic feature is the appearance of phosphatidylserine (PS) on the cell surface, which can be determined by an annexin-V binding assay. As shown in Fig. 4C, after 18 h of exposure to triterpenes, around 70–80% of the cells treated with 25–50 µM of erythrodiol and 40–60% of the cells treated with 50–100 µM of uvaol, displayed annexin-V binding. Taken together, these results suggest that alcoholic triterpenes trigger apoptotic cell death in 1321N1 astrocytes.

### Erythrodiol and uvaol trigger JNK activation on 1321N1 cells

The stress activated c-Jun N-terminal kinase (JNK) pathway is known to be involved in the regulation of apoptotic death in most cellular types [13]. Therefore, we explored whether JNK were activated in triterpene-treated cells, through an *in vitro* kinase assay.

**Figure 5:**
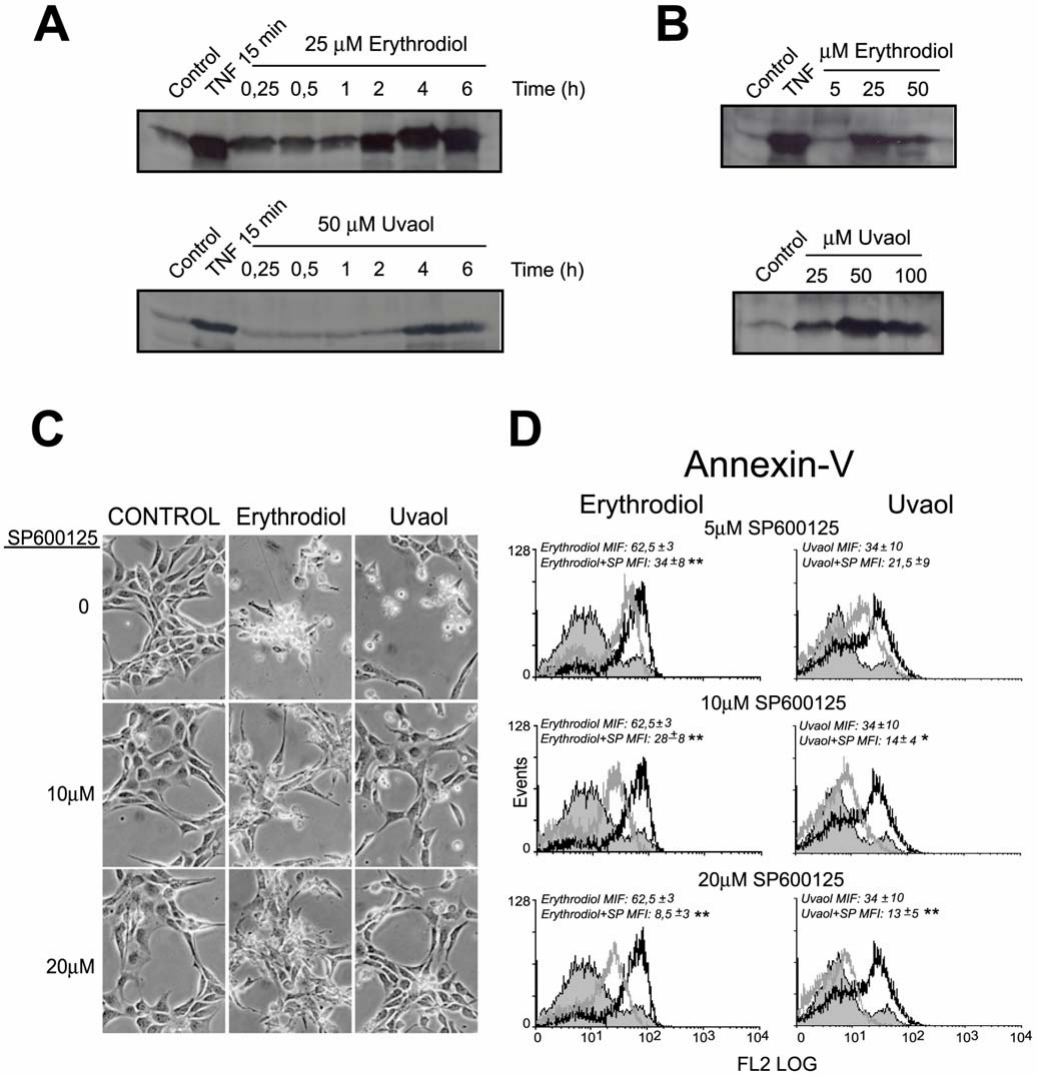
Effect of triterpenic diols in JNK activation. Cells were stimulated with 25 µM erythrodiol or 50 µM uvaol at the indicated times (A), or with different doses of erythrodiol or uvaol for 4 h (B), and assayed for an *in vitro* JNK-kinase assay as described in Materials and Methods. Exposure to 200 U/ml of TNF for 15 min was used as positive control. Results are representative of four separate experiments. Cells were exposed to different doses of SP600125 in the presence of 25 µM erythrodiol or 50 µM uvaol for 18 h. Then, the cells were analyzed by phase-contrast microscopy using a Nikon Eclipse TS100 microscope (×40; C) or labeled with annexin V–PE and analyzed by flow cytometry (D). In the histograms, cells obtained after triterpene treatment in the absence of the inhibitor (*open black* curves) are compared with cells treated in the presence of the inhibitor (*open gray* curves). *Gray solid* curves, resting control cells. *p<0.05, **p<0.01, vs triterpenes treated cells in the absence of the inhibitor.

As shown in Fig. 5A, both erythrodiol and uvaol modulate JNK activity. The maximum level of activation was observed after 4 h of stimulation with either drug. Dose-dependent studies (Fig. 5B) showed, once more, that cells were more sensitive to erythrodiol than to uvaol. The highest JNK activity was obtained at 25 µM of erythrodiol and at 50 µM of uvaol, which parallels with the concentrations required for apoptosis of this cell line.

Next, we studied the effect of the pharmacological inhibition of JNK on cell death events. 1321N1 cells were treated with different doses of the specific JNK inhibitor SP600125 before exposure to triterpenes, and then, analyzed under light microscopy (Fig. 5C) or stained with annexin-V for apoptosis evaluation (Fig. 5D). As shown in Fig. 5D, triterpene-induced cell death was markedly reversed by pretreatment with SP600125 in a concentration dependent manner. The cellular viability in control 1321N1 astrocytes was not affected by the inhibitor at the doses tested. These findings were confirmed by morphological examination of the cells under a contrast-phase microscope.

### Erythrodiol and uvaol cause reactive oxygen species accumulation

**Figure 6:**
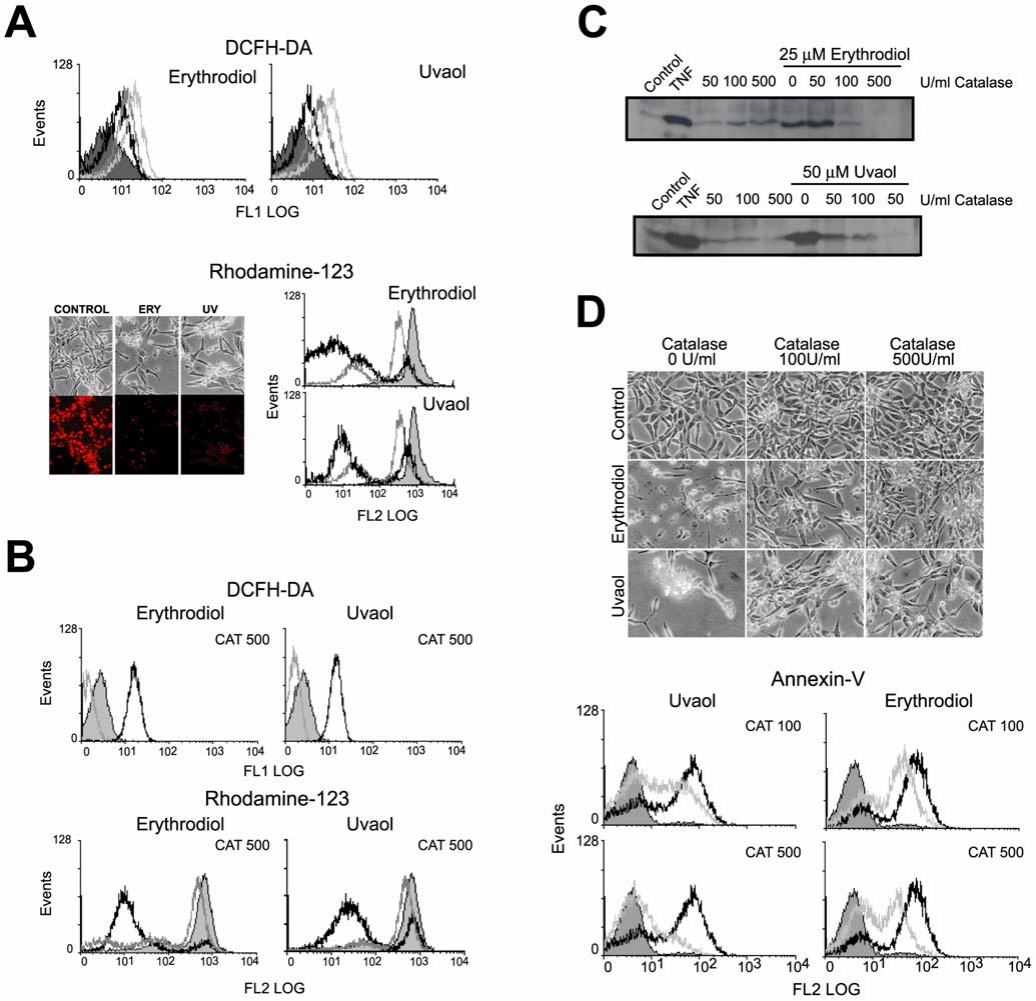
ROS-dependent JNK activation contributes to triterpenes-stimulated apoptosis. A, Analysis of ROS production and ΔΨ_m_ evaluation. Cells were treated with different doses of erythrodiol or uvaol for 30 min: 5 µM (*black empty* curve), 25 µM (*dark grey* empty curve) or 50 µM (*light gray empty* curve), and then stained with DCFH-DA (*upper histograms*), or treated with 25 µM erythrodiol or 50 µM uvaol for 6 h (*black empty* curve) or 18 h (*gray empty* curve) and staining with Rh123 (*lower histograms*). Intracellular ROS and ΔΨ_m_ was monitored by flow cytometry or under a fluorescence microscope (×40). B, Cells were preincubated with catalase, treated with 25 µM erythrodiol or 50 µM uvaol for 30 additional min and stained with DCFH-DA (*upper histograms*) or for 18 h and staining with Rd123 (*lower histograms*), and analyzed by flow cytometry. C, Cells were preincubated with different doses of catalase, treated with 25 µM erythrodiol or 50 µM uvaol for 4 h and assayed for an *in vitro* JNK-kinase assay. Exposure to 200 U/ml of TNF for 15 min was used as positive control. D, Cells were preincubated with catalase, treated with 25 µM erythrodiol or 50 µM uvaol for 18 h, stained with annexin-V-PE and analyzed under light microscope (×40) or by flow cytometry. In the histograms, cells obtained after triterpene treatment in the absence of the antioxidant (*open black* curves) are compared with cells treated in the presence of the antioxidant (*open gray* curves). In all the histograms, the *solid grey* curve represent the resting/control cells. The results are representative of three independent experiments.

Next, we asked whether erythrodiol or uvaol treatment were associated with changes in intracellular ROS levels. To examine this possibility, cells were loaded with the permeable and redox-sensitive dye, DCFH-DA and ROS production was assessed in the absence or presence of 5–50 µM of either erythrodiol or uvaol. In Fig. 6A *upper pannel*, the results demonstrate a dose-dependent increase in DCF fluorescence at 30 min of triterpene exposure. The mean fluorescence intensity (MFI) was 3.1 in non-treated cells, while after erythrodiol treatment was 5.3 at 5 µM, 10.1 at 25 µM, 15.1 at 50 µM and 14.85 at 100 µM. Similar MFI were obtained from uvaol-exposed 1321N1 cells. RNS production was also examined using the DAF-FM dye, but no oxidation was observed at any condition (data not shown).

It has been suggested that ROS overproduction leads to a reduction in the mitochondrial membrane potential (ΔΨ_m_) as well as mitochondrial dysfunction. Therefore, to detect these changes of ΔΨ_m_, the specific fluorescent probe Rhodamine 123 (Rh 123) was used (Fig. 6A *lower panel*). Rh 123 is a dye whose uptake and retention into the mitochondrial matrix depends on the membrane potential. Compared to untreated control cells, exposure of the cells to 25 µM erythrodiol or 50 µM uvaol for 6 or 18 h caused a disruption of ΔΨ_m_, as evidenced by a shift to the left in the fluorescence curves. Figure 6A *lower panel*, shows that both treatments caused a significant MFI decreased (p<0.001), for erythrodiol it dropped to 334.8±13,5 at 6 h and 184±33 at 18 h, and for uvaol was 278.7±10.5 at 6 h and 96.5±23 at 18 h, as compared with 929±31 in untreated control cells. These findings were confirmed by examination of the cells under a fluorescence microscope. Control cells were stained extensively with Rh123, whereas the triterpene-treated cells were less stained or not stained at all.

In addition, triterpene-induced reduction in ΔΨ_m_ was completely abrogated by catalase pretreatment (Fig. 6B). The sum of these results suggests that erythrodiol or uvaol induce ΔΨ_m_ dissipation in an antioxidant-sensitive pathway and it indicates that triterpene-mediated generation of ROS causes the reduction of ΔΨ_m_.

### ROS induced by triterpenic alcohols mediate JNK activation and apoptosis

To determine whether the pro-oxidant effects of triterpenes participate in erythrodiol- or uvaol-induced JNK activation and apoptosis, astrocytoma cells were pretreated with different concentrations of the ROS-scavenging enzyme, catalase. First, we determined the ability of this antioxidant enzyme to reduce the DCF fluorescence on triterpene-treated cells. As shown in Fig. 6B, the accumulation of intracellular ROS induced by erythrodiol or uvaol was completely abolished in the presence of 100 U/ml of catalase. Then, to find out whether oxidative stress was responsible for the activation of JNK, an *in vitro* kinase assay was performed with cells that were treated with different concentrations of the scavenger enzyme before exposure to erythrodiol or uvaol. The results showed that triterpenes-induced JNK activation was abrogated by catalase in a dose-dependent manner and the maximal effect was obtained at 500 U/ml. (Fig. 6C).

Finally, to further characterize the apoptotic response induced by the alcoholic triterpenes, the effect of catalase on the triterpene-induced cell death was determined morphologically under contrast-phase microscopy and by FACS analysis after annexin-V labelling (Fig. 6D). The results showed that the presence of the scavenger enzyme reduced in a dose-dependent manner the cell surface staining and attenuated the morphological changes elicited by triterpenes in 1321N1 cells. Therefore, the percentage of annexin V-PE-positive cells observed in erythrodiol- and uvaol-treated astrocytes decreased dramaticaly in the presence of 500 U/ml catalase, 70% vs. 43%, (p<0.01), and 60% vs. 18% (p<0.05), respectively, confirming the involvement of ROS in the process under study.

### Erythrodiol and uvaol upregulate some proteins of the TNF/TNFR family in 1321N1 cells

**Figure 7:**
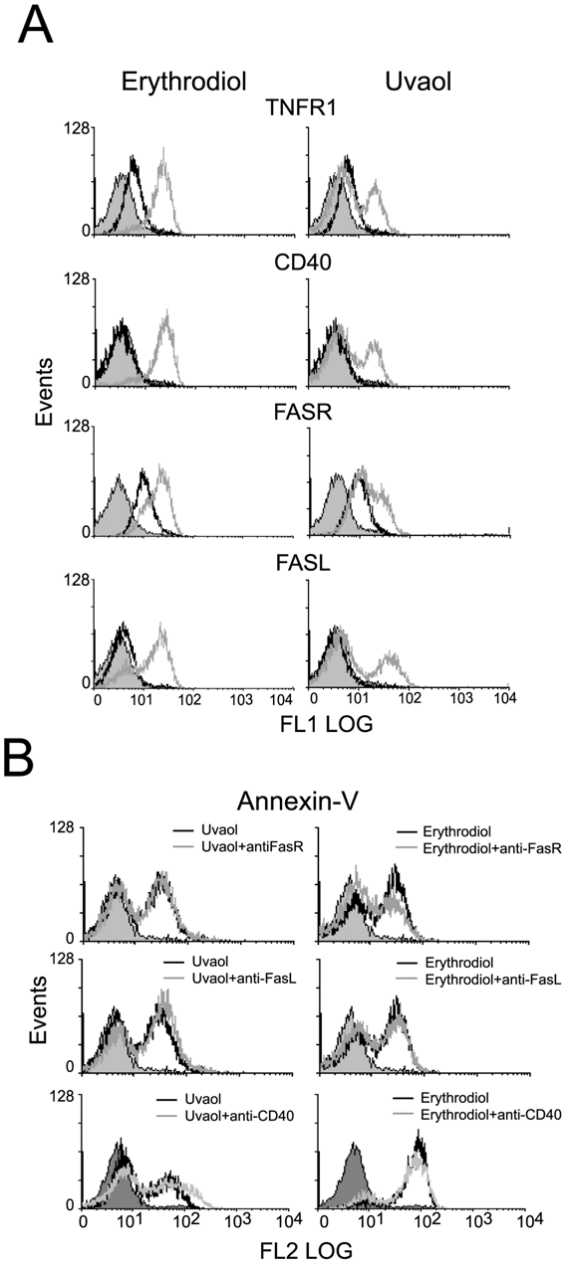
Effect of triterpenic diols on the expression and activity of death receptor/ligand systems. A, The expression of TNFR1, CD40, Fas and FasL was determined by flow cytometry, in 1321N1 cell exposed to 25 µM erythrodiol or 50 µM uvaol for 18 h. Histograms represent one experiment out of three. *Solid gray* curves represent unspecific binding; *empty black* curves, cells cultured in the absence of treatment (control); and *empty gray* curves, triterpene-treated cells. B, Cells were treated with 25 µM erythrodiol or 50 µM uvaol, in absence (*black empty* curve) or presence (*grey* empty curve) of anti-CD40, anti-Fas or anti FasL antibodies for 18 h. Afterward, the cells were labeled with annexin-V PE and analyzed by flow cytometry.

Possible pathways for erythrodiol- and uvaol-regulation of the cellular apoptosis threshold include the modulation of death receptor/ligand systems. Therefore, TNFR1, Fas, and FasL expression were measured by using flow cytometry analysis. As shown in Fig. 7A, a high proportion of 1321N1 cells constitutively expressed cell-surface TNFR1 and Fas, and the presence of 25 µM erythrodiol or 50 µM uvaol for 18 h was sufficient to markedly increase its cell surface expression. In addition, although these cells constitutively lack expression of FasL, we found it up-regulated upon triterpenes treatment. Similar results were also observed for CD40, another member of the TNFR superfamily.

Next, we wondered whether these death systems might account for a part of the mechanism of the triterpenic diols-induced apoptosis. As shown in Fig. 7B, we found that the presence of neutralizing anti-Fas or anti-FasL mAbs failed to significantly protect astrocytoma cells from the triterpenes-apoptotic effects, measured using annexin V-PE and flow cytometric analysis. Similarly, we found that CD40 ligation neither promoted annexin-V binding in resting 1321N1 astrocytes (data not shown), nor co-incubation with erythrodiol or uvaol affected the apoptotic response elicited by triterpenes in these cells. Therefore, we determined that these systems didn't play a role in transducing the apoptotic signals triggered by erythrodiol or uvaol.

### Apoptosis induced by treatment with erythrodiol or uvaol is a general event in cancer cell lines

**Figure 8:**
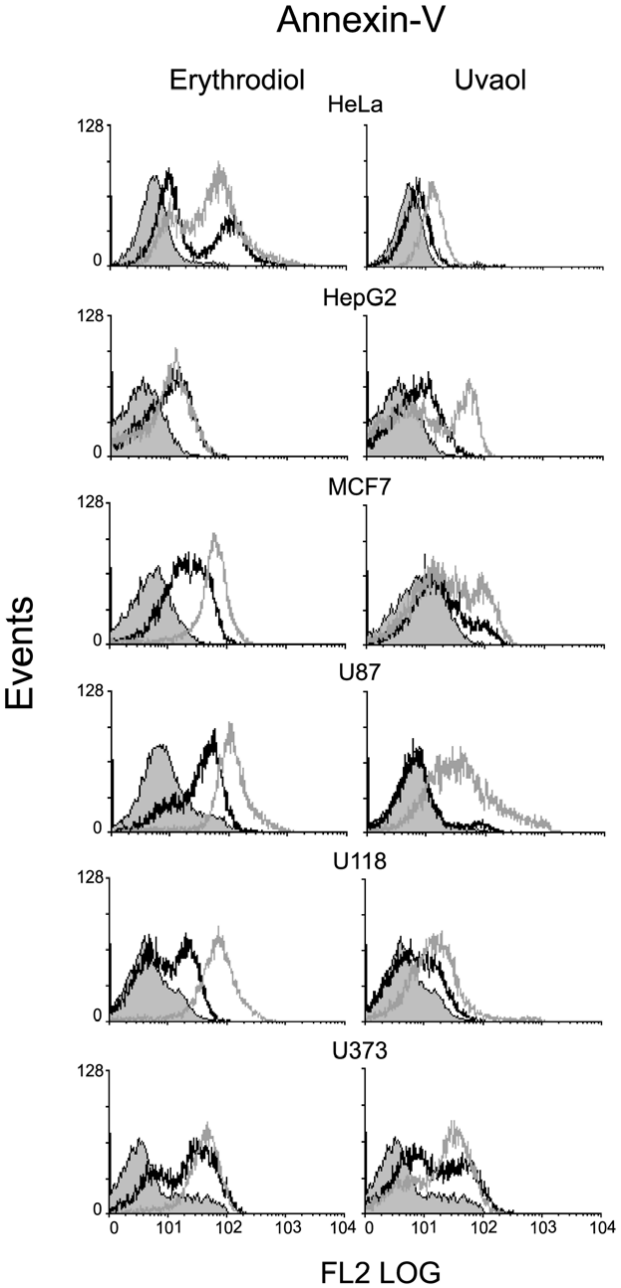
Effect of triterpenic diols on different tumor cell types. The indicated cell types were treated without or with 25 µM (*black empty* curve) or 50 µM (*grey* empty curve) of erythrodiol or uvaol for 24 h and stained with annexin-V PE. *Solid gray* curves represent labeling of resting/control cells.

To know whether the apoptotic response induced by treatment with either erythrodiol or uvaol was cell-specific or a general phenomenon, we performed the annexin-V binding assay in other human cancer cells independently of their p53 status, including, MCF7, HepG2, HeLa and three gliomas such as U373, U118 and U87. After 18 h exposure to 25–50 µM of erythrodiol or uvaol, all cell types under study bound annexin-V to a significantly higher degree than control-untreated cells, indicating that all of them underwent programmed cell death. The extent of the effects observed varied from cell to cell, and in all cases cells were substantially more sensitive to erythrodiol than to uvaol (Fig. 8).

## Discussion

Promising new molecules, especially those from natural origins, are being examined as therapeutic agents, since natural compounds are considered safe, as they are derived from commonly consumed foodstuff. Many reports have shown the anti-proliferative properties of triterpenes against multiple tumoral cells, being the acidic ones the most frequently studied [33]–[36]. In fact, to improve their activities, synthetic analogs of oleanolic acid have been developed and actually are under clinical evaluation as anti-tumoral therapeutic agents for hematologic malignancies. Surprisingly, in spite of the lack of effective treatments for brain tumors, little attention has been paid to these lipophilic agents capable of crossing the BBB [16]–[18]. In this study, we evaluated on astrocytoma cells the antitumoral activity of two alcoholic triterpenes, erythrodiol and its isomer uvaol, as well as their actions on some key players of the apoptotic response.

First of all, we show that both triterpenic diols are potent inhibitors of 1321N1 cells proliferation in a time- and dose-dependent manner. We also observe that this growth- inhibiting activity is associated with the induction of apoptosis. Treatment of 1321N1 cells with either erythrodiol or uvaol results in the appearance of apoptosis-specific hallmarks, such as redistribution of cells into the subdiploid phase of the cell cycle, translocation of phosphatidylserine to the outer leaflet of the cellular membrane, fragmentation of nuclei, and, production of ROS, which are accompanied by the fall in ΔΨ_m_. In addition, astrocytes undergo morphological changes normally associated with cell injury, indicating widespread alterations of the cytoskeletal framework. Cytoskeletal elements known to be reorganized during apoptosis include: actin microfilaments, microtubules, and intermediate filaments [39]. Thus, both by phase-contrast and fluorescence microscopy, we have shown that triterpenes cause retraction, rounding and shrinking of cultured cells, and elicit actin and vimentin filaments rearrangement: microfilaments and intermediary filaments dissolve, and an amorphous and condensed pattern is observed. Furthermore, cells show a significant reduction in the ability to both adhere and spread, and an altered expression levels of the standard form of CD44 is also observed, especially in erythrodiol treated cells. It is well known that the communication cell-cell and cell-extracellular matrix is facilitated by molecules like CD44, and a direct relationship between CD44 expression, actin filaments and the level of cell malignancy and resistance to cell death-inducing stimuli has been established [40], [41]. Therefore, the decrease in CD44 expression induced by triterpenic diols treatment may be one of the potential mechanisms coupled to the regulation of 1321N1 cell anchorage and death.

On the other hand, upon erythrodiol or uvaol treatment we also observe an upregulation of some members of the immunoglobulin super family, such as VCAM and ICAM that, at first sight, is not consistent with the antiadhesive and antiinflamatory effects of triterpenes. However, these receptors require clustering and association with actin-based structures for functional activity [42–44]; and it has been previously shown that disruption of the actin cytoskeleton not only influences cell surface distribution, but also increased surface expression of the ICAM-1 protein [45]. This means, that cytoskeletal disruption induced by triterpenes may alter the surface distribution of adhesion molecules, impeding their clustering, which eventually may result in reduced adhesion of 1321N1 cells regardless of their expression levels.

Interestingly, all these features also mimic those observed during a process named anoikis, a programmed cell death induced by the loss of anchorage. In fact, changes in cell shape and cytoskeleton might be considered a potential cause of anoikis. Cells adhere to natural extracellular matrices, via integrin-dependent interactions, as well as non-integrin-recognizable matrices such as poly-l-lysine. According to many studies, these interactions play an important role in survival and suppression of anoikis [46], [47]. In our study, the attachment of 1321N1 cells to poly-l-lysine matrix suppresses the apoptotic nuclear morphology induced after erythrodiol exposure. However, uvaol treatment either under adherent or standard conditions yields the same results, cells with condensed and fragmented nuclei. For this reason, we suggest the possibility that erythrodiol, but not uvaol, might induce cell death by anoikis in 1321N1 astrocytes. This is an interesting question for future studies, especially if we consider anoikis as a barrier to metastasis.

Extensive and recent studies about the functional properties of this pro-apoptotic family of drugs have suggested modulation of different key molecules as being responsible for its actions, depending on the triterpene and the cellular type. Thus, it has been reported for the synthetic oleanane CDDO-Me, modulation of Akt, NF-kB and Notch1 on gliomas [48] and JNK-mediated DR up-regulation on lung cancer cells [49]. On the other hand, betulinic acid has been demonstrated to promote degradation of the transcription factors Sp1, Sp3, and Sp4 on prostate cancer cells [50], while on melanoma cells it elicits a transient activation of the EGFR/ERK/AKT pathway [51] or promotes sequential effects on ROS accumulation and caspases activation on glioma cells [52]. In addition, endometrial cancer cells exposed to ursolic acid showed an inhibition of the PI3K/Akt and MAPK cascades [53], whereas JNK- and p38 kinase-mediated mitochondrial pathways participate in the apoptosis induced by echinocystic acid in HepG2 cells [54]. In this perspective, the present study shows that triterpene diols-induced JNK activation is mediated by ROS imbalance. It is likely that the main ROS formed is hydrogen peroxide, because addition of exogenous catalase suppresses 1321N1-DCF fluorescence, while superoxide dismutase fails to do so (data not shown). Moreover, pretreatment with catalase abrogates perturbance of ΔΨ_m_, JNK activation and rescues astrocytoma cells from triterpene-induced damage, indicating that ROS is the up-regulator of JNK activation during triterpene dialcohols-induced apoptosis. Interestingly, further investigations of mitochondria-associated apoptotic events also indicate involvement of caspase-3. Thus, the ratio of cells with activated caspase-3, rise from 6.5±4% in untreated cells to 31.3±4%, (p<0.05), and 23±4.8%, (p<0.05), upon 6 h of 25 µM ERY or 50 µM UV treatment, respectively (data not sown). However, the apoptotic response induced by other triterpenes such as betulinic acid, although also involve ROS generation upstream of the activated JNK, programmed cell death occurs independently of key caspases on melanoma cells [55].

In anticancer therapy, another key signaling mechanism known to play a role in the execution of apoptosis is the Fas/FasL system [56]. However, the role of this diad in brain tumors is controversial, since it has been observed that some tumors of the CNS expressing Fas/FasL are resistant to apoptosis induction [57]. In this regard, our study indicates that both markers are increased in erythrodiol- or uvaol-treated 1321N1 cells, as well as other proteins belonging to the TNFR superfamily such as TNFR1 or CD40. These findings are consistent with earlier data reported for others triterpene [58]. However, though 1321N1 cells are sensitive to Fas-mediated apoptosis (data not shown), this pathway has been discarded based on the inability of exogenously added anti-Fas/FasL blocking antibodies to attenuate triterpenic diols-induced apoptosis. Nevertheless, the possibility that a Fas/FasL suicidal interaction may take place in an intracellular compartment and, thus, is not accessible to exogenously added reagents can not be rejected [59]. In addition, our studies reveal that the up-regulated CD40 and TNFR1 are not required for triterpene-mediated apoptosis. Since, 1321N1 cells are neither sensitive to death after CD40 ligation, nor combining receptor ligation with exposure to triterpenic diols affect the response induced by the triterpene alone. Likewise, TNFR1 blockage does not affect triterpene cell death (data not shown). However, although these proteins are not mediators of the triterpenes-apoptotic pathway, they may be up-regulated to collaborate with the apoptotic program.

Furthermore, we find that erythrodiol and uvaol are able to induce apoptosis (in a dose-dependent manner) in other solid tumors cell lines irrespective of their p53 status, suggesting that their actions are not cell specific, but a generalized event.

In conclusion, our novel findings indicate that natural alcoholic triterpenes are powerful inhibitors of cell growth and efficient apoptotic killing agents and suggest that these processes are mediated by the activation of a ROS/JNK pathway.

## Materials and Methods

### Reagents

FITC-conjugated phalloidin, DAPI, PI, catalase, Rhodamine 123 and chemicals were from Sigma (St. Louis, MO). DCFH-DA and DAF-FM were from Molecular Probes (Eugene, OR). [^3^H]-Thymidine was from Amersham Biosciences (Little Chalfont, UK). SP600125 was from Cabiochem (San Diego, CA). Glutathione S-transferase (GST) fusion protein with amino acids 1–223 of the NH_2_-terminal portion of c-Jun protein was a gift from Dr. C. Caelles (IRB, Barcelona). For blocking experiments, the neutralizing mAbs NOK-2 (anti-FasL) (Pharmingen, San Diego, CA, USA) and SM1/23 (anti-Fas) (Alexis, Grünberg, Germany) were used at a concentration of 10 µg/ml.

### Erythrodiol and Uvaol

Erythrodiol and uvaol were from Extrasynthesis (Genay, France). Triterpenes solutions at 25 mM in EtOH were stored at −20°C and diluted to the final concentration in fresh media before each experiment. The final EtOH concentration used did not exceed 0.5%, so as not to affect the cellular responses. Structures are shown in Figure 1. For analytical assessment of purity and identity, erythrodiol and uvaol were converted into their trimethylsilyl (TMS) derivatives with TMSIM (N-Trimethylsilylimidazole), the preferred reagent for the silylation of the all hydroxyl groups, followed of GC/MS analysis [60], [61].

#### Conditions for GC-ion-trap-MS analysis

The GC-ion-trap-MS experiments were performed using a Finnigan Trace-GC 2000 gas chromatograph coupled to a Polaris-Q Ion trap mass spectrometer (ThermoFinnigan, Austin, TX, USA). The column used was a Zebron ZB-5 ms (Phenomenex, Torrance, CA, USA) fused silica capillary column (30 m long×0.25 mm i.d×0.25 m film thickness). The GC conditions included helium as carrier gas at 1 mL min^−1^ in constant flow mode. The initial temperature of 105°C was kept for 1 min, then raised to 290°C at a rate of 10°C/min and hold for 20 min. Injector temperature was 290°C and samples were inyected in split mode .

#### The MS operating conditions were as follows

ion source and transfer line temperatures were 200 and 290°C, respectively. The electron energy was 70 eV, resolution was unit and the emission current was 250 µA.

The Xcalibur version 1.4 software was used for data acquisition and processing of the results. Mass spectral identification was done by matching with those of the Wiley MS database and comparing the spectra to the literature.

Mass spectra of TMS derivatives of erythrodiol and uvaol showed a common fragmentation pattern: is spite the molecular ion is minimal or not present, it can be easily deduced by presence of loss of methyl group [M–CH_3_ ] at m/z 571, tipycal of silylated compounds, and a intense neutral loss of the TMSiOH group at m/z 496 that represent the base peak of the spectra of either compounds.

### Cell lines and cultures

The human astrocytoma cell lines 1321N1, U87 MG (U87), U181 MG (U181) and U373 MG (U373) were a gift from Dr J. Brown (UCSD, USA), Dr M. Guzman, (UCM, Spain), and Dr M. Izquierdo (UAM, Spain), respectively. The human carcinoma cell lines: breast MCF-7, cervix HeLa and hepatoma HepG2, were a gift from Dr. J.L. Bos (UMC, The Netherlands).

Cell lines were cultured under standard conditions in DMEM supplemented with 10% FCS as described [37].

### Cell morphological studies

Astrocytes were seeded on 16 mm glass coverslips. After triterpenoid treatment, cells were fixed with 3.7% formaldehyde, permeabilized in PBS containing 0.1% Triton X-100 and examined for morphological changes. In addition, the cells were incubated at 37°C in the dark for 30 min, with FITC-phalloidin (1∶200) and anti-vimentin antibody (1∶200) followed by incubation with Alexa 546-conjugated goat anti-mouse IgG antibody to visualize F-actin and vimentin. Cellular nuclei were detected by incubation with 1 µg/ml DAPI in the dark for 5 min. Thereafter, cells were washed with PBS and images were captured with either a Nikon Eclipse TS100 or a Nikon Eclipse 80*i* inverted fluorescence microscope using 20×, 40× or 60× objective lenses.

### Cell cycle analysis

Cells were seeded at the density of 5×10^5^ in 25-cm^2^ flasks. After 24 h, they were treated with or without different doses of triterpenes for 18 h, washed twice with cold PBS, and fixed with 70% ethanol. Then, RNA was removed by digestion with RNase A at room temperature, and cells were analyzed on an EPICS XL cytofluorometer (Beckman Coulter, Spain) after propidium iodide labelling. Data analysis was performed using WinMDI 2.7 software.

### Cell proliferation

Cell proliferation was evaluated with a [^3^H]-thymidine uptake assay [38]. Quiescent cells were treated with 5% FCS in the presence of 0, 1, 5, 25 or 50 µM of erythrodiol or uvaol for 24 h. Cells were then pulsed with 0.5 µCi [^3^H]-thymidine/well for 4 h before harvesting and the radioactivity incorporated was measured by liquid scintillation counting. Numerical data are expressed as mean±S.D. of three experiments, each performed in triplicate.

### Assays for ROS/RNS Production, and Mitochondrial Transmembrane Potential

The intracellular generation of ROS/RNS was measured using the cell-permeable probes 2′,7′-dichlorofluorescein diacetate (DCFH-DA) and 4-Amino-5-methylamino-2′,7′-difluorofluorescein diacetate (DAF-FM diacetate). Briefly, cells in 25-cm^2^ flasks were treated for 30 min with 1 µM DCFH-DA or DAF-FM and stimulated 30 min with 25 µM of erythrodiol, 50 µM of uvaol, or EtOH. Then, cells were harvested, washed in PBS, and analyzed by flow cytometry. In some experiments, cells were treated for 30 min with the indicated dose of catalase before incubation with the triterpenes.

To measure the mitochondrial transmembrane potential, cells were treated with the cationic dye Rh123. Cells in 25-cm^2^ flasks were treated with 25 µM of erythrodiol, 50 µM of uvaol or EtOH for 6 or 18 h. Then, Rh123 was directly added to the culture medium to a final concentration of 150 nM. Cells were harvested and analyzed for fluorescence intensity by flow cytometry or fluorescence microscopy.

### Flow Cytometric Analysis of Apoptotic Cells

After exposure to the indicated doses of erythrodiol or uvaol for 18 h the apoptotic cell death was determined using an Annexin V-PE Apoptosis Detection Assay, as described [33]. Briefly, cells were incubated in binding buffer with R-phycoerythrin- annexin-V antibody for 5 min, followed by flow cytometric analysis. In some experiments, cells were pretreated for 30 min with different doses of either catalase or SP600125.

### Flow Cytometry of Membrane Proteins

1321N1 cells were assayed for CD44, VCAM, ICAM, TNFR, CD40, Fas, and FasL expression. For this purpose, 5×10^6^ cells/dish were treated with the agonists for 18 h. Then, the cells were collected, suspended in PBS supplemented with 1% BSA and incubated with 10 µg/ml of : anti-human CD44 (A3D8), CD40 (5C3), ICAM (HA58), TNFR1 (MAB), VCAM mAbs; 1 µg/mL of FasL NOK-1 mAb, or 500 ng/mL of Fas DX2 mAb (BD Biosciences Pharmingen) for 1 h at 4°C. After washing, goat anti-mouse IgG-FITC conjugate (Sigma) 1∶100 was added and incubated for 30 minutes at 4°C. Subsequently, cells were washed and analyzed by flow cytometry. Positive cells were estimated using an isotype-matched control negative Ab at an equivalent concentration. Data were analyzed using WinMDI 2.7 software.

### Assay of JNK Activity

JNK assays were performed using GST-c-Jun as substrate, as described [34]. The substrate GST-c-Jun was expressed in bacteria *E. coli* XL-1 Blue using a pGEX-2T plasmid and purified with glutathione-agarose beads. The cytosolic extracts for the kinase assay were obtained from the lysis of 5×10^6^ cells. After centrifugation, the supernatant was mixed with 10 µg of GST- c-Jun protein and glutathione agarose beads. The mixture was incubated at 4°C for 3–5 h. Phosphorylated GST-c-Jun was resolved by 10% SDS/PAGE and immunodetected by Western blot using rabbit phosphospecific c-Jun (Ser^63^) antibody (Santa Cruz Biotechnology Inc, Santa Cruz, Calif).

### Statistical Analysis

Differences among the various treatment groups were determined by analysis of variance (ANOVA) followed by the Bonferroni's multiple comparison test with the GraphPad Prism Version 4 software (San Diego, CA). *P*-values lower that 0.05 were considered significant.

## Supporting Information

Figure S1 GS-MS chromatograms. GS-MS chromatograms. Gas Chromatography chromatograms in full scan mode of TMS derivatives of uvaol (A) and erythrodiol (C). Single peaks with retention times at 30.7 and 29.7 minutes, respectively, indicated that the preparation was >99% pure Gas chromatography ion-trap mass spectra of TMS derivatives of uvaol (B) and erythrodiol (D)
(0.93 MB TIF)
