# Supplementary figures and images for: Natural Triterpenic Diols Promote Apoptosis in Astrocytoma Cells through ROS-Mediated Mitochondrial Depolarization and JNK Activation
Source: PLoS One. 2009 Jun 22;4(6):e5975. doi: 10.1371/journal.pone.0005975 (PMC2695006; doi:10.1371/journal.pone.0005975)

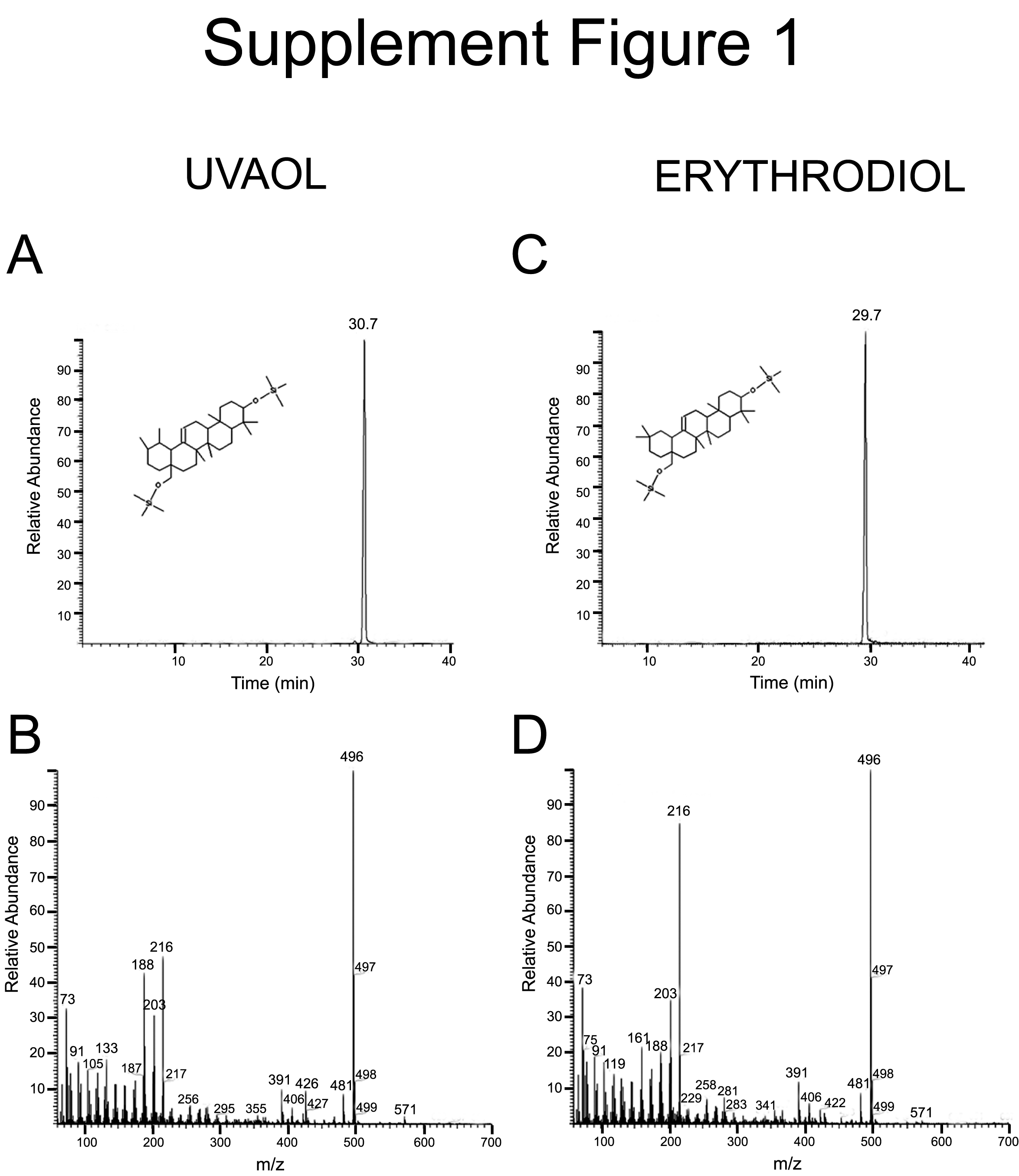

Supplement: Figure S1 — GS-MS chromatograms. GS-MS chromatograms. Gas Chromatography chromatograms in full scan mode of TMS derivatives of uvaol (A) and erythrodiol (C). Single peaks with retention times at 30.7 and 29.7 minutes, respectively, indicated that the preparation was >99% pure Gas chromatography ion-trap mass spectra of TMS derivatives of uvaol (B) and erythrodiol (D) (0.93 MB TIF) [file pone.0005975.s001.tif]
